# Exosome derived from stem cell: A promising therapeutics for wound healing

**DOI:** 10.3389/fphar.2022.957771

**Published:** 2022-08-08

**Authors:** Hui Lv, Hanxiao Liu, Ting Sun, Han Wang, Xiao Zhang, Wei Xu

**Affiliations:** Department of Clinical Pharmacy, The First Affiliated Hospital of Shandong First Medical University & Shandong Provincial Qianfoshan Hospital, Jinan, China

**Keywords:** exosome, stem cell, wound healing, therapeutics, wound

## Abstract

A wound occurs when the epidermis and dermis of the skin are damaged internally and externally. The traditional wound healing method is unsatisfactory, which will prolong the treatment time and increase the treatment cost, which brings economic and psychological burdens to patients. Therefore, there is an urgent need for a new method to accelerate wound healing. As a cell-free therapy, exosome derived from stem cell (EdSC) offers new possibilities for wound healing. EdSC is the smallest extracellular vesicle secreted by stem cells with diameters of 30–150 nm and a lipid bilayer structure. Previous studies have found that EdSC can participate in and promote almost all stages of wound healing, including regulating inflammatory cells; improving activation of fibroblasts, keratinocytes, and endothelial cells; and adjusting the ratio of collagen Ⅰ and Ⅲ. We reviewed the relevant knowledge of wounds; summarized the biogenesis, isolation, and identification of exosomes; and clarified the pharmacological role of exosomes in promoting wound healing. This review provides knowledge support for the pharmacological study of exosomes.

## Introduction

The skin is the largest multifunctional organ in the body. It can prevent bacterial invasion and resist chemical and physical assaults by forming a strong barrier between the organism and the environment (Proksch et al., 2008; Wang et al., 2019). A wound is a disruption of normal anatomic structure and function because of internal and external breakage of the epidermis and dermis and occurs when the skin is torn and burned and has pressure ulcer and diabetes ulcer (Reinke and Sorg, 2012; Kanji and Das, 2017; Ayavoo et al., 2021). A prompt and suitable wound healing is necessary for the repair of functional tissues as well as disordered structures after an injury (Gurtner et al., 2008; Ayavoo et al., 2021). Normal wound healing is a sophisticated and dynamic activity that involves many physiological activities, such as inflammation, cell proliferation, and extracellular matrix remodeling (Wang et al., 2018). Chronic wounds fail to heal orderly and timely through normal healing mechanisms (Velnar et al., 2009), which are characterized by long healing time and scar hyperplasia.

According to the statistics, chronic wounds affect more than 6 million people. It is anticipated that the number of people with chronic wounds will increase among our growing elderly and diabetes population (Powers et al., 2016). Chronic nonhealing wounds will make patients experience serious pain and become physically anxious (Järbrink et al., 2017), which would bring strong pressure on society (Järbrink et al., 2017). Therefore, the development of novel technologies and practices in the best practice clinical management of chronic wounds is imperative to diminish the possible burdens on the health and economy of the society and optimize the healthcare management for this prospective silent pandemic.

Conventional wound care methods include wound dressings (Han and Ceilley, 2017), skin substitutes (Dai et al., 2020), and growth factors (Shpichka et al., 2019). However, the drawbacks of these methods such as long healing time, immune rejection, high cost, and easy infection (Goodarzi et al., 2018; Vu et al., 2021) limit their application. In recent years, stem cells for wound healing have become one of their most important tools because of their strong self-renewal and differentiation ability (Nourian Dehkordi et al., 2019). Studies have confirmed that the effect of stem cell therapy has to do with the paracrine effect mediated by stem cell secretory factor exosomes (Yang J. et al., 2020). The exosome is the smallest extracellular vesicle, which is released into the extracellular environment after the fusion of late endosomes with the plasma membrane (Hessvik and Llorente, 2018). Exosome derived from stem cell (EdSC) is secreted by stem cells, which can transfer functional cargos such as proteins, DNA, and RNA from donor cells to the recipient cells (Nikfarjam et al., 2020; Gurung et al., 2021) and mediate intercellular communication to promote the activities of wound healing–related cells, such as fibroblasts and keratinocytes (Arishe et al., 2021). In this review, we reported the biogenesis, isolation, and identification of exosomes; elaborated on the mechanism of exosomes promoting wound healing; and discussed the clinical trials of exosomes in the treatment of wound healing.

## Representative therapeutics for wound healing

**FIGURE 1:**
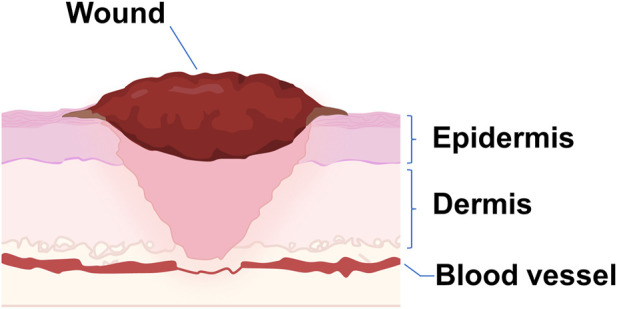
Schematical illustration of a wound. A wound occurs when the dermis and epidermis of the skin, as well as blood vessels, are damaged.

Wounds are disruption of normal anatomic structure and function because of internal and external breakage of the epidermis and dermis (Reinke and Sorg, 2012; Kanji and Das, 2017; Ayavoo et al., 2021) (Figure 1). Current strategies for wound healing include wound dressings (Han and Ceilley, 2017), skin substitutes (Dai et al., 2020), and growth factors (Shpichka et al., 2019).

### Wound dressings

Wound dressing is a sterile pad that is used in direct contact with the injury (Tang et al., 2021), which can keep a local moist environment around the wound, protect the wound from micro-organisms, and sustain good gas transmission (Kamoun et al., 2017). Common wound dressings include cotton gauze, human amniotic membrane, and polysaccharide-based factors (Zeng et al., 2018). However, traditional wound dressings are limited in providing a proper sterile environment for wounds (Farahani and Shafiee, 2021).

### Skin substitutes

Skin substitutes are heterogenous biomaterials that can provide a substitute for the extracellular matrix to accelerate the healing process of wounds (Auger et al., 2009; Dai et al., 2020). According to different sources, it can be divided into allografts of human origin and xenografts of animal origin (FerreiraCastropaggiaro et al., 2011). This way provides a physical barrier from bacteria (Wei et al., 2022) and trauma and can keep a moist microenvironment in the wound bed (Dai et al., 2020), but allogenic skin grafts have the risk of immune rejection (Iy and Al-Rubaiy, 2009).

### Growth factors

Growth factors can affect the microenvironment in the wound bed when released. (Dolati et al., 2020), such as promoting intercellular communication, including endothelial cells and fibroblasts (Werner and Grose, 2003). Although the direct application of growth factors is beneficial to wound healing, it also has certain limitations. For example, under the action of protein hydrolases, growth factors will degrade quickly (Golchin et al., 2018).

## Recent emerging novel tool: exosome derived from stem cell

Stem cells play an important role in wound healing and skin regeneration because of their strong self-renewal and differentiation ability (Mazini et al., 2020; Guillamat-Prats, 2021; Jo et al., 2021). The main tissue sources of stem cells for wound healing and skin regeneration include fat, bone marrow, and umbilical cord. In the process of wound treatment, using stem cells can close the wound early and reduce scar formation (Isakson et al., 2015; Guillamat-Prats, 2021). Of note, many studies have reported the effect of mesenchymal stem cells (MSCs) in wounds and regenerative medicine through their paracrine factors such as exosomes.

The emergence of EdSC offers new possibilities for wound healing. As a cell-free therapy, EdSC overcomes the limitations of stem cells. EdSC therapy is easy to use and time-saving and has low immune rejection (Ha et al., 2020). Studies have found that EdSC can induce benefits in nearly all phases of wound healing. For instance, it can inhibit inflammation, control immune responses, and promote cell proliferation and angiogenesis (He et al., 2019; Chen Md et al., 2021).

### Biogenesis of exosome derived from stem cell

**FIGURE 2:**
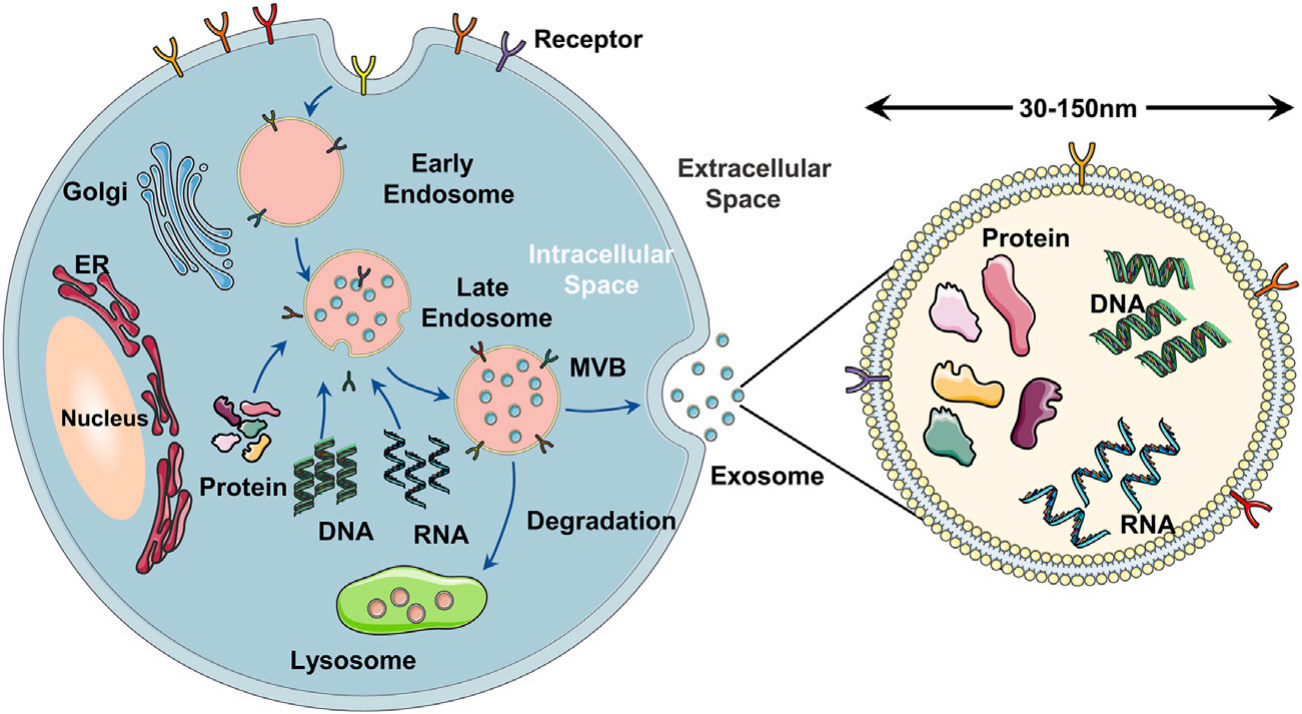
Schematical illustration of the exosome biogenesis. **(A)** the cell membrane forms early endosomes in the form of endocytosis. **(B)** the early endosomes mature into MVB containing exosomes after further acidification. **(C)** the MVB fuses with the cell membrane and releases exosomes into the extracellular space in the form of exocytosis.

Exosomes are a subset of extracellular vesicles that are secreted by the majority of the types of cell-like dendritic cells, T cells, stem cells, and a variety of cancer cells (Isaac et al., 2021). According to the biogenesis and size, extracellular vesicles can be divided into exosomes, microvesicles, and apoptotic bodies (Crescitelli et al., 2013; Thakur et al., 2021). The diameter of exosomes is 30–150 nm, which is the smallest extracellular vesicle (Raposo and Stoorvogel, 2013). The release of exosomes occurs via three major steps (Figure 2): 1) Cell membrane invaginates to form primary endosomes, and the early endosomes are acidified into late endosomes. 2) The late endosomes bud inward to form a multivesicular body (MVB) (Tiwari A. et al., 2021). 3) Exosomes are released into the extracellular environment after the fusion of MVB and plasma membrane by exocytosis (Huotari and Helenius, 2011; Van Niel et al., 2018; Gurunathan et al., 2021). The exosomes secreted by stem cells such as adipose-derived stem cells and umbilical cord MSCs are EdSCs (Yu et al., 2014). EdSC contains many biomolecules of donor stem cells, such as DNA, RNA, nucleic acid, lipids, metabolites, and cytosolic (Kalluri and Lebleu, 2020; Gurung et al., 2021). Surrounded by lipid bilayers, EdSC can regulate cell–cell communication by transferring a lot of functional biomolecules to recipient cells (Tran et al., 2020; An et al., 2021; Arishe et al., 2021). As the main paracrine factors of stem cells, many studies established that EdSCs are also involved in the immune response (Hodge et al., 2021; Dai et al., 2022), cancer prevention and treatment (Jiang et al., 2022; Nik Nabil et al., 2022), antigen presentation (You et al., 2021; Zheng et al., 2022), angiogenesis (Yang et al., 2021), drug delivery (Riau et al., 2019; Ding et al., 2022; Rao et al., 2022), and inflammation (Shen et al., 2021). Multiple pharmacological effects make EdSC attract much attention in enhancing skin wound healing (Las Heras et al., 2020; Al Gailani et al., 2022; Dong et al., 2022; Opoku-Damoah et al., 2022).

### Technologies for isolating exosome derived from stem cell

The isolation of pure EdSC is critical to understanding its mechanism and application to wound healing. Conventional methods include ultracentrifugation-based techniques, size-based techniques, and immunoaffinity capture-based techniques (Wu et al., 2019).

(1) For ultracentrifugation-based techniques: Ultracentrifugation-based techniques are known as the “gold standard” for EdSC isolation technology (Yang D. et al., 2020), which is according to the difference in size and density of each constituent in mixture solution (Livshits et al., 2015; Saad et al., 2021). Ultracentrifugation can be divided into density gradient ultracentrifugation and differential ultracentrifugation (Konoshenko et al., 2018; Gurunathan et al., 2019). Differential ultracentrifugation is easy to operate and low in cost (Zhao R. et al., 2021). However, compared with differential centrifugation, the purity of EdSC isolated by density gradient centrifugation is higher (Shirejini and Inci, 2021; Tarasov et al., 2021).
(2) For size-based techniques: Size exclusion chromatography is a typical technology for separation based on the size of exosomes. The sample containing exosomes flows through a stationary phase column filled with a porous matrix. The sample molecules smaller than the pore size can diffuse into the matrix and need a longer time to pass through the column, whereas large molecules get eluted faster (Tiwari S. et al., 2021). This method cannot distinguish EdSC and microvesicles of the same size, and the yield of exosomes is low. However, it is quick, easy, and cheap, and the isolated EdSCs are uniform in size and intact biophysically and functionally (Heydari et al., 2021; Purghe et al., 2021; Saad et al., 2021).
(3) For immunoaffinity capture-based techniques: Immunoaffinity-based isolation strategies use antibodies that were embedded with different materials such as magnetic beads (Kandimalla et al., 2021) to target the specific surface antigens of exosomes (Li S. et al., 2021). Then, antibody-recognized exosomes are captured (Alzhrani et al., 2021). This method can evidently increase the purity of EdSC and save time and samples of isolation (Li S. et al., 2021). However, the defect of this method is that it is not suitable for the isolation of large sample volumes (Alzhrani et al., 2021). Moreover, it only works with cell-free samples and isolates EdSC with low yield (Fu et al., 2019).

### Tools for identifying exosome derived from stem cell

Once EdSCs are isolated, they need to be further quantified and analyzed (Li S. et al., 2021). According to the International Society of Extracellular Vesicles, the identification techniques of EdSCs can be based on morphology, size, and specific proteins on the surface of exosomes (Thery et al., 2018), such as electron microscope, nanoparticle tracking analysis (NTA), and western blot.

(1) For electron microscope: Electron microscopy techniques have been widely used to detect the morphology and size of EdSC (Alzhrani et al., 2021). It mainly includes a cryo-electron microscope and a transmission electron microscope (TME). In TME, two electron beams pass through the samples and are subsequently collected and magnified (Liu Q. et al., 2021). However, EdSCs show a saucer-like structure under TME (Zhao R. et al., 2021). Many researchers attribute this phenomenon to the collapse of samples caused by drying during sample processing (Cizmar and Yuana, 2017). Unlike TME, EdSCs detected using a cryo-electron microscope are round (Jin et al., 2021). This technique is now widely used since the destruction of the sample is avoided.
(2) For NTA: NTA can identify the dimension as well as the concentration of EdSC (Zara et al., 2020). The Brownian motion of suspended particles and light scattering are the basic principles of NTA (Pelissier Vatter et al., 2020; Zhao R. et al., 2021). By viewing in the mind each very small bit through image observations using either distributed widely light or gave out fluorescence, NTA measures the Brownian motion of person EdSC and connects it to a very small bit size (Carnino et al., 2019; Alzhrani et al., 2021). The advantages of this method include high detection sensitivity, easy sample preparation, fast analysis speed, and suitability for a large number of samples (Zara et al., 2020; Singh et al., 2021). However, this technique is accompanied by the problems of poor sensitivity, low efficiency of sorting targeted EdSC, and poor reproducibility (Jin et al., 2021).
(3) For western blot: Western blot identifies EdSC based on the specific proteins on the surface. Specific proteins (such as CD9, CD81, and CD63) are separated by electrophoresis and then combined with the corresponding antibodies (Zhao et al., 2019; Jalaludin et al., 2021). This identification method has high sensitivity and specificity (Singh et al., 2021). However, workflow is prolonged (Singh et al., 2021).

## A promising therapeutics, exosome derived from stem cell, for wound healing

**FIGURE 3:**
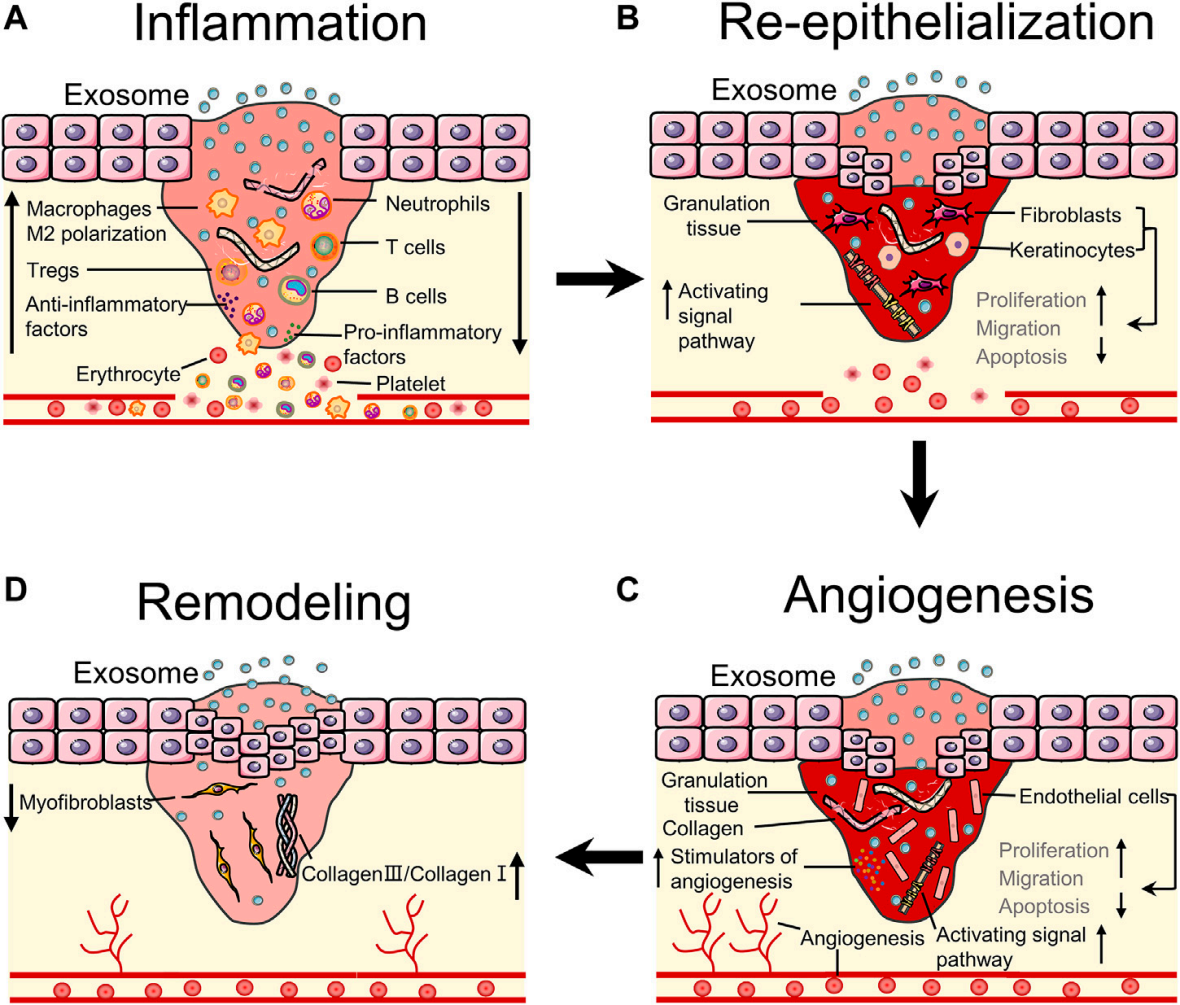
Bioeffects of stem cells derived exosomes on wound healing. **(A)** EdSC can inhibit inflammation by regulating the number of inflammatory cells and the polarization of macrophages. **(B)** EdSC can promote re-epithelialization by increasing the activity of fibroblasts as well as keratinocytes and activating pathways. **(C)** EdSC can improve angiogenesis by stimulating the release of angiogenic factors and regulating the activity of endothelial cells. **(D)** EdSC can improve tissue remodeling by regulating the ratio of collagen and myofibroblast differentiation.

EdSC can be administered to the wound through intravenous injection and subcutaneous injection (Hu et al., 2016). After administration of EdSC to the wound site, it can induce benefits in almost all stages of wound healing, such as inhibiting inflammation by controlling immune cells, accelerating wound closure and angiogenesis by promoting proliferation and migration of cells, and reducing scar formation by regulating the proportion of collagen (Li D. et al., 2018; Wei et al., 2021). This part mainly discusses the delivery systems and mechanisms of EdSC that promote wound healing (Figure 3).

### Exosome derived from stem cell delivery systems for wound healing

The most common way of administration of EdSC is intravenous injection. For instance, delivering EdSC to the wound site of mice through intravenous injection can stimulate the activities of fibroblasts, to accelerate wound healing (Hu et al., 2016). Subcutaneous injection is another delivery method of EdSC for wound healing. Liu et al. injected melatonin-stimulated EdSCs subcutaneously into the wound and found that EdSCs enhance diabetic wound healing by regulating the polarization of macrophage M1 phenotype to M2 phenotype through targeting PTEN/AKT pathway (Liu et al., 2020). Although the method of direct injection seems more efficient, it is highly invasive (Kosaka et al., 2012; Akbari et al., 2020). Although injection is simple and effective, it can limit EdSC therapeutic function because the clearance rate of this route is relatively rapid (Takahashi et al., 2013). In recent years, many researchers have combined EdSC with hydrogel to prolong the efficacy time and improve the stability of EdSC, to accelerate wound healing. As a new dressing, a hydrogel is a three-dimensional structure formed by physical or chemical crosslinking between hydrophilic polymer chains (Zhao Y. et al., 2021; Ma and Wu, 2022; Safari et al., 2022). Hydrogels can load EdSC by absorbing a large amount of solution containing EdSC because of the hydrophilic functional groups in polymers (Xu et al., 2018; Golchin et al., 2022). Applying the EdSC-loaded hydrogel dressing to the wound bed, the hydrogel network can control the release concentration and time of EdSC and increase the moisture content of the wound (Kim et al., 2017; Shafei et al., 2020). It maintains a good microenvironment in the wound bed that supports the activities of loaded cells to accelerate wound healing (Kim et al., 2017; Riha et al., 2021). Hence, hydrogels are utilized as desirable therapeutic agents for EdSC on wound healing.

### Inflammation

Inflammation is characterized by removing debris and preventing infection through activation and recruitment of resident immune cells, such as neutrophils, mast cells, and eosinophils (Wang et al., 2018). Accumulating evidence suggests that EdSCs inhibit the process of inflammation in various pathways. Studies have confirmed that MSC-exosomes can block the infiltration of neutrophils and reduce the number of neutrophils in wounds to prevent excessive inflammation (Li et al., 2016; Shojaati et al., 2019). In addition, MSC-exosomes from different sources reduce pro-inflammatory factors such as IL-1, IL-6, TNF-ɑ, IFN-γ, IL-17, TNF-ɑ, and IL-1β along with increase anti-inflammatory factors such as IL-10, TSG-6, IL-4, and TGF-β in wounds to accelerate the inflammatory process (Liu et al., 2014; Chen et al., 2016; Nojehdehi et al., 2018; Li K. L. et al., 2021; Shen et al., 2021). In the later stage of inflammatory, pro-inflammatory M1 macrophages are transformed into anti-inflammatory M2 macrophages, which can activate fibroblasts, keratinocytes, and endothelial cells to promote re-epithelialization as well as angiogenic processes (Rani and Ritter, 2016; Sorg et al., 2017). For one thing, Xu et al. found that under LPS stimulation, exosomes from BMSCs resulted in an increase in M2 macrophage polarization and a decrease in M1 macrophage polarization (Xu et al., 2019). For another thing, some researchers have found that stem cell–derived exosomes from different sources can also induce the polarization of macrophage M1 phenotype to M2 phenotype through various biological pathways. For example, exosomes derived from MSCs stimulated by melatonin have been shown to enhance diabetic wound healing by targeting the PTEN/AKT pathway (Liu et al., 2020). Meanwhile, Khare et al. demonstrated that BMSC-derived exosomes promote the inflammation process by decreasing the proliferation and activation of B cells (Khare et al., 2018). In addition, EdSC also can suppress inflammatory T cell proliferation (Blazquez et al., 2014; Di Trapani et al., 2016; Monguio-Tortajada et al., 2017), activate T cells into T regulatory cells (Chen et al., 2016), and increase the number and proliferation of Tregs (Del Fattore et al., 2015; Chen et al., 2016; Du et al., 2018; Nojehdehi et al., 2018; Zhang et al., 2018; Riazifar et al., 2019; Li K. L. et al., 2021). In the immune system, dendritic cells are the cells that present antigens that can enhance T cell proliferation (Xie et al., 2020). Reis et al. found that dendritic cells can be inhibited by exosome treatment, which indirectly inhibits T cell activity (Reis et al., 2018). Therefore, all evidence suggests that SCdEs have anti-inflammatory potential and the ability to prevent excessive inflammation.

### Re-epithelialization

In the phase of re-epithelialization, fibroblasts proliferate and migrate in large numbers and produce and deposit ECM to form granulation tissues that replace initial fibrin clots and repair tissue losses (Wilkinson and Hardman, 2020). EdSCs were readily taken up by fibroblasts that stimulate cell activity to promote wound healing (Hu et al., 2016; Chen Md et al., 2021; Tutuianu et al., 2021). As a scaffold, granulation tissue supports migration as well as the proliferation of wound cells and promotes new angiogenesis. Meanwhile, fibroblasts can stimulate keratinocytes by secreting keratinocyte-derived growth factors, which can undergo a partial epithelial–mesenchymal transition (Sorg et al., 2017). Then, keratinocytes proliferate and migrate toward the wound center until contact with reverse cells stops (Han and Ceilley, 2017). EdSC can promote wound healing by regulating keratinocyte and fibroblast characteristics and enhancing re-epithelialization. In addition, ADSC-derived exosomes enhance keratinocyte activities by activating Wnt/β-catenin signaling, AKT/HIF-1alpha signaling, or AKT pathway to promote wound healing (Ma et al., 2019; Zhang et al., 2020). According to Chen et al., highly expressed microRNA-21 in ADSC-exosomes can increase the MMP-9 expression via the PI3K/AKT to promote the activity of the keratinocytes (Yang C. et al., 2020). Moreover, ADSC-exosomes inhibited miR-19b expression via lncRNA H19 (H19) and activated the Wnt/β-catenin pathway using upregulated SPY-related high-mobility group box 9 (SOX9), resulting in enhanced human skin fibroblast function (Miao et al., 2021; Qian et al., 2021). Human ADSC-exosomes contain lncRNA MALAT1, which is capable of increasing fibroblast migration in the dermis (Cooper et al., 2018). Jeffrey et al. suggested that CD63^+^ exosomes from BMSCs contribute to the transport exterior Wnt3a signal to recipient cells significantly, thereby promoting fibroblast and endothelial functions (Mcbride et al., 2017). In addition, the study also found that umbilical cord–derived MSC (uMSC)-exosomes contain microRNAs such as miR-21, miR-23a, and miR-125b, which can suppress the differentiation of fibroblasts into myofibroblasts formation via inhibiting collagen deposition (Mcbride et al., 2017) to accelerated re-epithelialization (Fang et al., 2016; Li D. et al., 2021). Taken together, the role of exosomes in promoting re-epithelialization is mainly achieved by enhancing the function of keratinocytes and fibroblasts.

### Angiogenesis

Angiogenesis is another important process in the proliferative phase. Promoting angiogenesis is the main factor for stem cell–derived exosomes to promote wound healing. Angiogenesis provides oxygen, blood supply, and metabolic pathways for wound healing. Hypoxic environment after injury has induced the release of fibroblast growth factor 2 and vascular endothelial growth factor A. This stimulates vascular endothelial cell proliferation to build new blood vessels. TutuiaNu et al. demonstrated that exosomes stimulated endothelial cell migration via scratch test assay (Tutuianu et al., 2021). In addition, stimulators of angiogenesis such as angiopoietin-2 (Ang-2) and endothelin (ET-1) were found in EdSC (Tutuianu et al., 2021). Meanwhile, exosomes derived from human uMSCs can improve angiogenesis by delivering angiopoietin-2 to promote wound healing (Liu J. et al., 2021). Wounds in the feet of diabetic rats treated with exosomes from ADSCs overexpressing Nrf2 exhibited reduced ulcer area, granulation tissue formation, enhanced growth factor expression, and increased angiogenesis (Li X. et al., 2018). In recent years, it is reported that embryonic stem cell–derived exosomes can activate Nrf2 to improve endothelial senescence (Chen B. et al., 2019). Some studies have found that stem cell–derived exosomes can transfer RNA or protein, such as miR-125a (Liang et al., 2016), miR-31 (Kang et al., 2016), miR-21 (An et al., 2019), and DMBT1 protein (Chen et al., 2018), to promote angiogenesis for wound healing. Ding et al. demonstrated that exosomes from human BMSCs can stimulate angiogenesis by activating the PI3K/AKT signaling pathway *in vitro* (Ding et al., 2019). Signaling pathways with similar efficacy include AKT/eNOS pathway (Yu et al., 2020) and Wnt4/β-Catenin pathway (Zhang et al., 2015). As mentioned above, stem cell–derived exosomes accelerate wound healing via promoting angiogenesis.

### Remodeling

In the remodeling stage, the primary task is to reduce scar formation. Uncontrolled accumulation of myofibroblasts that contract the wound and excessive proportion of collagen Ⅲ in the wound lead to scar formation (Zeng and Liu, 2021). In granulation tissue, collagen Ⅰ replaced collagen Ⅲ gradually to promote scar-free repair. In recent years, some studies observed the effects of exosomes on matrix remodeling. Liu et al. injected exosomes secreted by human ADSCs intravenously into murine incisional wounds and found that ADSC-exosomes can reconstruct ECM in wound bed by regulating the proportion of collagen-like type Ⅲ to type Ⅰ and reduce scar formation by regulating differentiation of fibroblast (Wang et al., 2017; Wang et al., 2021). MiR-192-5p expressing exosomes derived from human ADSCs can mitigate hypertrophic scar fibrosis by modulating the smad pathway. Its performance in wound healing is attenuated collagen deposition, transdifferentiation of fibroblasts to myofibroblasts, and formation of hypertrophic scars (Li Y. et al., 2021). In addition, microRNAs enriched in epidermal stem cell–derived exosomes (EPSC-exos) include miR-16, let-7a, miR-425-5p, and miR-142-3p (Duan et al., 2020). EPSC-exos-specific microRNAs, such as miR-425-5p and miR-142-3p, can reduce the TGF-β1 expression in dermal fibroblasts to inhibit myofibroblast differentiation (Duan et al., 2020). Furthermore, EdSC suppressed scar formation by reducing collagen deposition and regulating inflammation (Jiang et al., 2020). The findings of li et al. indicated that ADSCs-exosomes facilitate scar-free healing by enhancing the characteristics of fibroblasts (Hu et al., 2016; 2020). EdSC increased collagen Ⅰ and Ⅲ production through systemic administration at the initial stage of wound healing, whereas EdSC may inhibit collagen expression to reduce scar formation in the late stage (Hu et al., 2016). Taken together, EdSC can reduce scar formation by regulating the proportion of collagen.

## Clinical applications of exosomes in wound healing therapy

The therapeutic potential of stem cells and EdSC for wound treatment have been conducted in various kinds of animal studies. It demonstrated that stem cell injection not only effectively suppresses inflammation but also enhances re-epithelialization and angiogenesis and reduces scar formation, which can promote the repair of skin wounds through secretion of stem cell-like EdSC. However, the structure and physiology of animal skin are different from those of human skin. Therefore, it is important to understand the mechanism of EdSC in human skin wounds.

From clinicaltrials.gov, we retrieved several clinical trials about wound healing treated with MSCs, but only two have exosomes. Using stem cells to treat burns as early as 2005, this study demonstrates that BM-MSC therapy is safe, which promoted angiogenesis and accelerated granulation tissue formation (Rasulov et al., 2005). Another completed clinical trial, which began in 2019, used stem cell–conditioned medium containing exosomes or microbubbles to treat chronic ulcerative wounds and found that conditioned medium stem cells can improve skin ulcer healing as an additional growth factor for the first time (NCT04134676). In addition, we retrieved a clinical trial initiated by Kumamoto University—the effect of plasma-derived exosomes on skin wound healing. Participants’ wounds were treated with plasma rich in exosomes for 28 days to evaluate the effect of exosomes on skin wound healing (NCT0256526). This study found that compared with normal subjects, patients with chronic wounds such as skin ulcers had significantly lower levels of serum exosomes. In conclusion, the results of existing clinical trials show that exosomes can accelerate skin wound healing.

Similar to wound treatment, we can retrieve 258 clinical trials when the key word is exosome, such as for periodontitis (NCT04270006), melanoma (NCT02310451), chronic low back pain (NCT04849429), knee osteoarthritis (NCT05060107), and COVID-19 (NCT05216562). With the increasing number of clinical trials on the therapeutic effect of exosomes, it is believed that exosomes will come out as a therapeutic drug as soon as possible.

## Conclusion

EdSCs are small in size and efficient, have low immune rejection, and have special physiological and biological functions, which have significant advantages for the treatment of wounds. With scientific and technological progress, a deeper understanding of EdSC, and the treatment of related diseases in the medical field, interdisciplinary integration will complement and enhance the application of EdSC in various fields. For example, combining the advantages of exosomes as carriers with advanced design methods of nano-medicine can establish a nano-treatment platform based on EdSC. In addition, understanding the interaction between exosomes and other organelles is helpful to better understand the process of disease (Chen Q. et al., 2019; Chen et al., 2020). The most difficult component of the research of exosomes is the inadequate number of exosomes meeting the application standards. In the near future, advances in the scaling-up technology for GMP-compliant exosome manufacturing will enhance the applications of exosomes for wound healing.
